# Barriers to accessing public cancer genomic data

**DOI:** 10.1038/s41597-019-0096-4

**Published:** 2019-06-20

**Authors:** Katrina Learned, Ann Durbin, Robert Currie, Ellen Towle Kephart, Holly C. Beale, Lauren M. Sanders, Jacob Pfeil, Theodore C. Goldstein, Sofie R. Salama, David Haussler, Olena Morozova Vaske, Isabel M. Bjork

**Affiliations:** UC Santa Cruz, 1156 High Street, Mailstop: Genomics Institute, Santa Cruz, CA 95064 USA

**Keywords:** Policy, Scientific community

## Abstract

Although increasingly recognized as critical to genomic research, genomic data sharing is hindered by an absence of standards regarding timing, patient privacy, use agreement standards, and data characterization and quality. Only after months of identifying, permissioning for use, committing to terms restricting use and sharing, downloading, and assessing quality, is it possible to know whether or not a dataset can be used. In this paper, we evaluate the barriers to data sharing based on the Treehouse experience and offer recommendations for use agreement standards, data characterization and metadata standardization to enhance data sharing and outcomes for all pediatric cancer patients.

Genomic data sharing is increasingly recognized as critical to genomic research. The National Institutes of Health (NIH), through its Genomic Data Sharing Policy, and other leading research funding agencies and journals now regularly require grantees and authors to make genomic data available to the research community, either post-publication or after an embargo period. For example, pediatric research foundations St. Baldrick’s Foundation and Alex’s Lemonade Stand Foundation have recently added data sharing as part of awardee reporting requirements. A 2017 study^1^ analyzed data sharing policies of 318 biomedical journals and found that 11.9% require data deposition and this requirement correlated with increased impact factor of the journal. Sharing of data can benefit all research, including translational research focused on human health. Unfortunately, accessing usable data from public repositories is challenging due to intersecting issues of data location, characterization, quality assessment, use approval and compliance, resulting in delays and unreasonable resource expenditures. Data sharing efforts are leading the charge. For example, NIH’s Data Commons is developing a cloud-based platform for storing and sharing digital data and software. The National Cancer Institute (NCI) Genomic Data Commons (GDC) provides a data repository that enables data sharing across cancer genomic studies and supports cancer genome programs, including The Cancer Genome Atlas (TCGA) and Therapeutically Applicable Research to Generate Effective Treatments (TARGET). Additional repositories for genomic data include Gabriella Miller Kids First Data Resource Center, Cavatica, St. Jude Cloud, and the Foundation Medicine Pediatric Portal. In this paper we identify the difficulties researchers face when attempting to access genomic data, and suggest solutions and recommendations to enable researchers everywhere to more readily use shared data to investigate and defeat cancer.

In fields that suffer from paucity of data and rarity of tumor types, such as pediatric cancer research, shared data are particularly helpful. Motivated by the observation that pediatric cancers have a lower and different mutation burden than adult cancers^2^, the Treehouse Childhood Cancer Initiative (Treehouse), a pediatric cancer-focused project at the University of California Santa Cruz Genomics Institute, is modeled on the idea that the genomic data of one sick child can be compared against a large quantity of genomic tumor-derived data from thousands of kids and adults, who also suffered from cancer, thereby informing treatment options. The more data that are explored by way of such cross-cancer comparisons (pan-cancer), the more robust the analysis^3^. We examine individual pediatric cancer tumor gene expression profiles against a growing compendium of 11,000+ tumor gene expression profiles. This compendium is publicly available at https://treehousegenomics.ucsc.edu^4^. It is a combination of clinical cases from partner institutions, including Stanford University, UC San Francisco, Children’s Hospital of Orange County, and British Columbia Children’s Hospital, and data from research studies, made public in various repositories for use by researchers. The process of acquiring data from public repositories, which make up the majority of the data in the Treehouse compendium, is the focus of this paper. A separate process governed by institution-specific data use agreements applies to clinical data from partner hospitals.

Of the 11,485 samples in the Treehouse compendium, 11,239 samples are from public repositories. The team began with TCGA data, primarily adult tumor data processed through the UCSC toil pipeline^5^, and has worked systematically to enhance its compendium of RNA sequencing (RNA-Seq) expression data by adding pediatric cancer data and data from underrepresented tumor types. Table 1 summarizes the data that make up the current version of the Treehouse compendium.

**Table 1:** Number of samples per dataset in the Treehouse compendium v9 (polyA+).

Dataset	Number of Samples
TCGA	9,806
TARGET	784
International Cancer Genome Consortium (ICGC), including 17 samples from Medulloblastoma Advanced Genomics International Consortium	210
St. Jude – Washington University Pediatric Cancer Genome Project (PCGP)	103
Children’s Brain Tumor Tissue Consortium (CBTTC)	29
Other public datasets of pediatric or rare adult cancer data	307
Treehouse Partner data (all pediatric)	246
**Compendium Total**	**11,485**

Public repository data mining includes at least five steps: (1) identifying the relevant data and location of the data, (2) obtaining data access, (3) downloading the data, (4) characterizing the data, and (5) assessing data quality. Each step has associated difficulties and can be time and budget fatiguing, and the progression from step to step is not linear. It is our experience that once data of interest are identified, it takes 5–6 months on average to obtain access to and prepare public genomic data for research use.

## Steps to Using Shared Data & Associated Challenges

### Step 1. Finding the data

Researchers seeking to use public data must comb websites and public repositories, e.g., Database of Genotypes and Phenotypes (dbGaP) and European Genome-Phenome Archive (EGA), search biomedical and life sciences journal literature, contact authors/researchers directly, and engage in time-consuming trial and error. It has been standard academic practice for researchers to withhold data until publication. At the time of publication, the data are typically submitted to public repositories in order to make possible access by other researchers. In practice, publications may come out long after completion of the research. Even after paper publication, researchers may postpone release of the underlying data until the publication of subsequent work containing some or all of the same data. The Treehouse team’s search for RNA-Seq data associated with a study reported in a September 2015 publication^6^ illustrates the difficulty of identifying what information is available and when. The publication referenced a potentially valuable dataset but did not provide the identifying information. Five months into a process that included many repeated publication and data searches, as well as requests to publication authors, we learned that the data described in the publication would not be made public until a second manuscript, with some data overlap, was published (which ultimately did not occur until 2 years after the first publication)^7^. By the time data are released, knowledgeable researchers and staff may have moved to new projects or positions, limiting follow-up questions regarding the data. Large consortia, common in genomic research, involve multiple parties, each with varied publication interests, timelines, and administrators, which may further complicate data release.

**Fig. 1:**
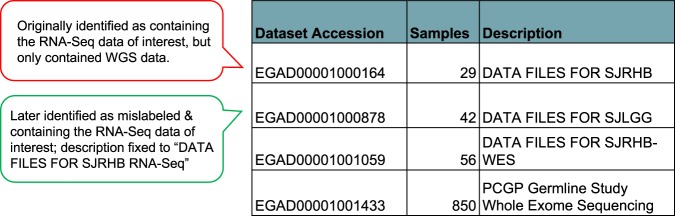
Simplified and annotated recreation of the original table of datasets in EGA study, EGAS00001000256.

Once data are deposited, mislabeling sometimes occurs, or, more often, the label omits location or specific name of the study/dataset necessary to track the data. Hours of reading papers and examining repository dataset descriptions can be required to match data described in a publication with a repository location, and researcher clarification may still be required. As illustration, a paper referenced an EGA study, EGAS00001000256, which contained four datasets from at least two papers, but had insufficient information to determine which dataset contained the RNA-seq data from the paper of interest. Figure 1 is an annotated and simplified recreation of the table of cryptically named datasets in EGAS00001000256 that we found on the study web page.

In response to our email requesting clarification, the author identified EGAD00001000164 as the relevant dataset. We applied for permission, which was granted a few months later. Upon download of the metadata, we discovered that the data were whole genome sequencing (WGS) data and not the RNA-Seq data our research required. Further follow up with the author revealed that the RNA-Seq data were actually in another dataset, EGAD00001000878, which had been incorrectly labeled. Ultimately, the description was corrected and we applied for permission. Unnecessary use of time and resources would have been avoided by the inclusion of standard descriptive language following the labeling, irrespective of human error in the labeling. In another instance, we were informed that potentially valuable RNA-Seq data were available, but were unable to locate the data through a repository or publication search. Contact with the paper’s authors revealed that the RNA-Seq data inadvertently had not been submitted, and the EGA study accession label referenced in the paper was incorrect. The Treehouse team has encountered at least four additional instances (phs000720.v2.p1, phs000768.v2.p1, EGAS00001002528, and EGAS00001002314) in which RNA-Seq data were included in the paper, but the RNA-Seq data were not present in the repository when we initially checked. Standard monitoring of repository information would avoid this error. Often, authors are not aware of data discrepancies until teams like Treehouse hunt the data. The combination of inconsistently named datasets, multiple data types, the practice of grouping datasets from multiple papers under a single study accession, and the absence of explanatory data descriptions makes the identification of target data within a database challenging.

### Step 2. Obtaining access

While data may be intended for public use, most are subject to controlled access, requiring researchers to describe their proposed use of the data and apply for access. On average, a straightforward application and approval process takes 2–3 months. In complicated instances, it has taken up to 6 months. Applications and the resulting contracts often have cumbersome data use, reporting and renewal requirements that often require multiple submissions and exchanges to clarify terms and purpose. At UC Santa Cruz, as with most research institutions, these permissions require multiple levels of internal review, including legal review by the University counsel, sometimes resulting in a lengthy process, particularly when there are associated requirements for data use. Obtaining access to samples that are available from international sources adds another layer of complexity, due to international law. In the case of data requests to a Canadian partner, the USA Patriot Act was viewed by Canadian colleagues as prohibiting download of raw data files from their site to ours. Approval for data access took over 6 months and required not only new agreements, but also approval from the Canadian Finance Ministry.

Most agreements grant access for one year, at the end of which a report summarizing research progress is required; for continued data access, yearly renewal requests are required. For instance, dbGap’s yearly report requires a list of publications, conferences and any intellectual property developed that include the relevant data. As the year of access starts from the date of approval, a research team with a number of approvals must juggle multiple reporting requirements and deadlines. Some sources, such as ICGC, require that each person touching the data is listed on the agreement and that the list be updated of any changes within 30 days. BC Cancer Agency requires a copy of any manuscript or other disclosure document 30 days in advance of submission of publication, and St. Jude requires a copy of any publication arising from the use of the data within 30 days of publication.

Additionally, clarity, completeness and reliability of available information can be opaque when trying to obtain access to data. For example, in September 2017, we sought access to xenograft data found in a 2017 Nature paper^8^, but could not apply for access until the data and descriptions of the datasets were posted, delaying our initial request until November 2017. That request was denied in January 2018, on the basis that all data would be made available on a cloud platform, which was not yet live. Although the cloud platform went live in April 2018, the 2017 xenograft data were still not available on the cloud platform as of March 2019.

### Step 3. Downloading primary genomic data

Software for securely downloading these genomic files is not standardized; each repository has its own custom suite of tools, which must be installed and configured. Even when the software is well-documented and user-friendly, the necessity of learning a new toolchain for each dataset is time consuming. The act of downloading data also is typically slow. Primary data files, in formats such as FASTQ or BAM files, are multiple gigabytes in size. In theory, high-speed scientific networks should be capable of delivering these files quickly; in practice, the speed is often throttled by high network usage, software bottlenecks on either server- or client-side, and other connection issues. Once the data are downloaded, they may need to be decrypted, or converted from a repository’s custom file format to the standardized format that downstream tools require.

### Step 4. Characterizing the data

Characterization is the process of determining which donor, sample, protocol, and clinical details each file downloaded corresponds to. This includes determining whether multiple data files are from one or more samples, donors, timepoints, or tissue-types, and identifying the type of sequencing data (as previously noted, data mislabeled as RNA-Seq data or ambiguously labeled have, on several occasions, turned out to be whole genome or whole exome data). As team experience has grown, and after wasted time and not insignificant cost, Treehouse has established internal protocols that require a separate assessment of metadata and file size prior to data download. Nonetheless, data download often reveals that the data are more, less, or different than anticipated based on the labeling.

Initial data identification efforts often do not provide the metadata information needed; it is only when downloaded that key attributes such as age, diagnosis, histology, stage of the tumor, and RNA-Seq methods become available. Unfortunately this information is often incomplete or error ridden, leading to *data characterization* challenges. Of the 23 datasets we have included in our compendium, 12 are from EGA, which offers little information about the data prior to permissioning. Usually only a brief summary and a title for the dataset is available. Once permission is granted, the sample name, sample accession, sample type, technology, and file type are made available. Only after download is additional information sometimes available and cross-referencing the publication for additional information is required. By contrast, the dbGaP repository includes a list of samples with many technical details about how the data were generated and often histology, prior to permissioning. When repositories provide several publications for a dataset, it falls on the user to read through each paper (and its supplements) to locate relevant information about the data that may be described in the publication. Often no publications are listed in the data repository. While we understand that respect for individual privacy presents legitimate characterization challenges, requiring restrictions on identifying age and sex in rare diseases, for instance, a standardized approach to data characterization that added core descriptions would eliminate core characterization barriers.

### Step 5. Data quality

Only after identifying, permissioning for use, and downloading the genomic and metadata, is it possible to assess the data quality. Low quality data (low read coverage or poorly formatted, which can cause our pipelines to fail to complete analysis) can be limited to individual data files or cover entire datasets. As noted, characterizing and assessing quality cannot be done in most cases until downloading has occurred. In our case, from June 2016 to August 2018 poor data quality resulted in over 166 samples downloaded, prepared and processed that were not usable due to low quality, costing approximately 350 project hours.

A serious concern is cost, and the potential dampening effect this resource drain will have on genomic research. When data providers require that their data only be accessed and processed on their preferred cloud platforms instead of allowing users to download the data, cost may become prohibitive. In the case of the St. Jude Children’s PCGP data, dating back to November 2017, we have been seeking access to four datasets, totaling over 150 pediatric samples. Those data now reside in the St. Jude Cloud along with the rest of the PCGP data. We have been granted permission to run our pipeline in the St. Jude Cloud to analyze the RNA-Seq data for just under 900 PCGP samples, but initial estimates were that doing so could cost in excess of $50,000 and would require technical changes to our pipeline. Ultimately, we reached an agreement with the platform company for reduced cost for this pilot phase. Overall, it took 12 months from our first request to receive access to the data; we are concerned about possible future costs for processing any new data that become available. We remain concerned about the broader community impact of the high technical and financial resource requirements involved in this method of data sharing.

In summary, obstacles to acquiring shared genomic data can result in a difficult, time-consuming and often discouraging quest. The current process taxes resources and disadvantages small, sparsely financed research projects. Increasingly, initiatives are promoting data sharing, including policy development. These include the NIH Genomic Data Sharing Policy, and sharing platforms for defined stakeholder groups, such as participants in a consortia, clinical, or government initiatives. Existing sharing platforms and resources, not specifically focused on genomic information include: the NCI Cancer Data Standards Registry and Repository (caDSR), which allows searching metadata from cancer clinical trials; NCI thesaurus (NCIt), which covers vocabulary for cancer research and clinical care; the coordinating centers for NCI projects such as the Cancer Immune Monitoring and Analysis Centers for NCI-sponsored immunotherapy trials; Coordinating Center for Cancer Systems Biology Consortium; and Human Tumor Atlas Network, which will include genomic characterization data from cancer samples and models. Several disease-specific foundations have also developed data portals containing genomic and clinical information for that disease, such as a portal for neurofibromatosis research. Still, clarity and enforcement questions remain, and efforts need to harmonize in a way that extends across stakeholder groups. We include here some suggestions for improving the practicalities of data sharing, with the aim of improving and democratizing data sharing among researchers everywhere.

## Suggestions for Improved Data Sharing

### Accelerating publication and data release

The peer review process is a cornerstone of science: a peer reviewed publication affirms that the research has been heavily vetted by experts in the field, undergone a comment and review process, and is therefore worthy of trust and reliance. However, it is also a lengthy process, often involving multiple revisions and resubmissions. When data are held back from public access until a peer reviewed paper is published, researchers may lose the opportunity to evaluate how those data inform their research. A delay in the release of data has the potential to delay advances in science. Manuscript archiving, which allows for pre-publication of the submitted version of a manuscript, enables the timely release of research data. It also allows researchers to show their research to the academic community and interested readers, ensuring that work is recognized and credit for new discoveries is not dependent on reviewer or editorial delay. Biology-focused archives such as bioRxiv are increasingly used in bioinformatics and genomics research, indicating that this may be growing in acceptance among new and established researchers. Faster release of data could accompany biobanking publications, subject to protections regarding attribution and use^9^.

### Requiring normalized repository descriptions and metrics

Normalized descriptions and metrics of existing genomic data that alert researchers to what data are available, before any requests or access has occurred, would save valuable researcher resources. We envision that enhanced descriptions of data content will serve to reduce the impact of human error, as the multiple fields will allow users to identify inconsistencies more readily.

Such key normalized descriptions and metrics for genomic sequencing data could include:

- number of uniquely mapped reads
- file sizes
- data type
- sequencing assay type
- relevant publications associated with each dataset
- study description
- minimum essential characteristics, such as age, sex, disease

A benchmarking exercise to assess whether any additional information is commonly needed by the research community, and the positive features of existing repositories, would benefit researchers around the globe.

### Standardization of metadata

Where possible, genomic data should be tagged with metadata to allow concurrent download of genomic and clinical data. Development of standardized minimum essential characteristics of metadata would improve consistency. Within some sharing platform initiatives, efforts have been made to harmonize and curate data. In cancer, the GDC, caDSR, and NCIt are federally funded efforts to standardize data; in the non-cancer space, HCA and NIA AMP-AD are good examples of efforts already underway. The challenge for the genomic data research community is to draw from these efforts to create broad-reaching metadata standards, allowing initiatives and platforms to come together with a unified approach that will allow research efforts to cross-pollinate and extend outward beyond individual stakeholder groups. A vital benefit of standardizing metadata language through commonly used data dictionaries is opening possibilities for machine learning applications to find and use data. Establishing a structured approach to findability, accessibility, interoperability and reusability, as defined by the FAIR Principles^10^ for data resources, will support research discovery globally.

As guidelines are developed to standardize metadata, a risk analysis of patient identification is required. Too often, well-intentioned researchers and institutional representatives apply overly inclusive protections in a broad-sweeping attempt to cover all potential risks, with the result that insufficient data are often disclosed, even in instances where such restrictions are not necessary. We recommend that guidelines are developed to assist researchers in determining where descriptors pose a genuine risk of identification so that exclusions to metadata disclosure are narrowly tailored to the risk presented. One solution is that in such narrowly tailored instances, generalized metadata can be provided that give broader, less identifiable information about certain characteristics, in particular age.

### Technical tool development

Standardized download processes and tools, and clear instructions, would ensure broader accessibility. Identification of existing data sources would be improved by the development of a search engine with the capability to mine all repositories. Recent initiatives such as DataMed^11^ are directed at addressing this need; a coordinated approach to facilitate capture of all data within developing mining tools is critical.

### Help/contact

Built-in notification systems to report errors or request clarification should encourage reporting of issues and errors by data requestors/users with the goal of improving information about the data. One possibility is to adopt a public and transparent issue tracking system to record data user issues regardless of repository, encouraging resolution of issues, and disseminating data user issues broadly. Additionally, repositories could adopt a “data release status” for tracking datasets for which the accession has already been published, so that users are informed of timing of data availability. In the longer term, such automation of user feedback could track use itself. Data sharing in the absence of usability or application is without research benefit. Unfortunately due to laboratory turnover, this change will have a limited impact and must supplement larger changes having to do with standardization of metadata.

### Reconsideration of data use agreements

Standardized applications with parallel terms and fixed reporting and review dates would reduce duplication and administrative time for research teams. A regular review of data use agreements could reconsider the use restrictions and the reasons behind them. Perhaps certain genomic data types, such as gene- and exon-level quantification information derived from tumor RNA-Seq data, which is minimally traceable to an individual, require different treatment from germline whole genome sequence data, which carry greater potential for traceability and triggers privacy or discrimination concerns. Risks vary with data type; access protections should be appropriately assigned according to data type. Equally, a clear assessment of risk requires consideration of the state of data. There is a need to distinguish between protections needed for access to the raw protected data and protections needed for sharing analysis results from the protected data. When the latter is sufficiently derivative as to eliminate traceability, the risk of sharing is de minimis. The development of guidelines that link data type and risk to access rules could eliminate unnecessary barriers.

### Integrating cost analysis and budget considerations in research funding

Insufficient attention is given to an important consequence of sharing initiatives: unanticipated increased or re-directed cost burdens. Not only does accessing shared data require personnel time and expertise, but it also includes direct costs. Downloading data incurs bandwidth egress costs on either the provider or receiver of the data. Sharing models that require researchers to use a designated cloud platform to compute the data and receive only the output of that compute avoid this bandwidth cost but can be quite costly in terms of compute charges, particularly when large data sets are used. Funders and policy-makers need to acknowledge that cloud-based sharing and computing platform solutions cost money, and in many cases the burden on the user may be insupportable. When cloud platforms are developed, a cost-use analysis must be done, and use of third party vendors, which is becoming increasingly common, needs to be weighed against the added financial burden this may pose on researchers. While cloud-based platforms offer technical solutions, they may exclude researchers who are not well funded, having the adverse effect that only well funded researchers and industry will be able to use this “shared data” resource. In parallel, implementing guidelines to improve metadata descriptions (pre-download/compute) will lessen research burdens of searching for relevant data, and will diminish download and compute costs because it will significantly reduce the number of instances of compute or download effort expenditure on data with unknown provenance. One concern is that costs will be transferred from data user to data provider, with the cost to standardize borne by data providers. A commitment to sharing in principle must mean a commitment in practice. When researchers deposit data, they must be willing to spend the time necessary to characterize the data and address follow-up questions from data users. We acknowledge that standardizing will push more costs on the data sharers. In order to prevent the de-incentivization of sharing and the delay of innovative research, additional research funds must be targeted to sharing efforts to realign the cost burden.

The extraordinary potential for shared genomic data to benefit human health is finding a voice through data sharing initiatives, spearheaded by leaders such as the NIH and realized through important initiatives such as the NIH Data Commons and Global Alliance for Genomics and Health (GA4GH). The Cancer Gene Trust is an example of the GA4GH’s work in this area. Yet, in our experience, many statements of data availability from researchers and repositories are not correct or exaggerated. For data that actually are available through a repository, the amount of work required to access those data is prohibitive to the end user, typically requiring expert administrative, legal, and scientific support. Such support is costly and not available to smaller projects with limited funding. We recognize that data sharing presents real challenges for researchers, data providers and repositories. In order not to retard research, we recommend that implementation of standardization, guidelines or harmonization efforts should occur in stages. For instance, it takes time to develop or change policy, and once new rules are in place, it takes time to effect the new requirements. We suggest that data sharing should continue to occur apace, with a reasonable implementation time provided that minimizes delay. Incentives from funders are required to galvanize implementation of new policy into practice. When funders provide money to support sharing enterprises, the cost-use analysis should be included in the proposal and budget for the new data support efforts so that user costs are controlled, either through subsidies to users, reductions for bulk requests, and/or sufficient third party funding such that third party vendors do not push their costs onto users. While some cost issues will be reduced as technology improves, research should not suffer while the market adjusts to new technologies or competition.

Through the development of shared guidelines around data use agreements, technical tool development, accelerated publication and data release, data set integration, and automated help tools, data sharing initiatives can actually be used by researchers on the ground to further precision medicine. In doing so, groundbreaking data sharing initiatives can be accessible to researchers everywhere, without discrimination that results from undue resource burdens on access. Taking this idea a step further, some researchers are calling for creators of pediatric cancer genomic resources to work together to create a real-time federated data-sharing system to facilitate pediatric cancer data sharing^12^. Particular consideration should be given for teams from smaller institutions with limited resources and to creating a mechanism to encourage the sharing of secondary data analysis by researchers who have successfully navigated access to these primary data resources. While data sharing efforts are receiving increasing attention and support, greater scrutiny of the details of data access from an accessing researcher’s point of view would stimulate shared data use to expand precision medicine research worldwide.
